# Supplementary material for: Structural Fluctuations of the Human Proteasome α7 Homo-Tetradecamer Double Ring Imply the Proteasomal α-Ring Assembly Mechanism
Source: Int J Mol Sci. 2021 Apr 26;22(9):4519. doi: 10.3390/ijms22094519 (PMC8123668; doi:10.3390/ijms22094519)
Supplement: Supplementary file 1 [file ijms-22-04519-s001.zip › ijms-1173437-supplementary/5-SupplementaryMaterials-v2/5-alpha7-ijms-supple-v2.pdf]

Supplementary Information

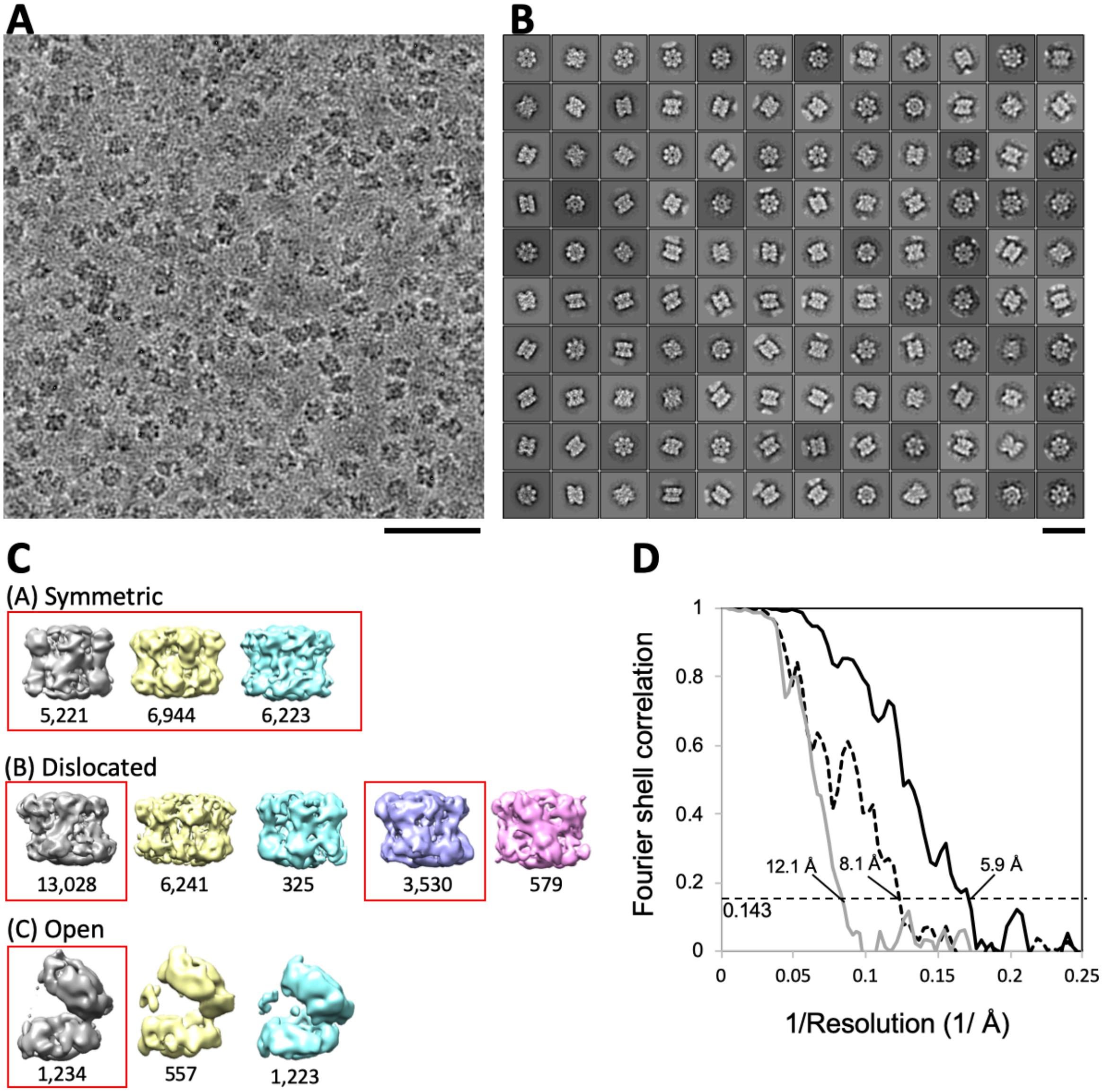

Supplementary Figure S1. Single particle cryo-EM data processing of the α7 homo-tetradecamer. **(**A) Representative micrograph of the α7 homo-tetradecamer. Scale bar, 500 Å. (B) Example of the 2D class average images. Scale bar, 200 Å. (C) 3D classifications of the symmetric, dislocated, and open types. The classes surrounded by red squares were used for the subsequent 3D refinement for each type. (D) Plots of the gold-standard Fourier shell correlations of the cryo-EM maps of the symmetric, dislocated, and open types indicated by black, dotted and gray lines, respectively. Based on the 0.143 criterion for comparing two independent datasets, the resolutions of symmetric, dislocated, and open types are estimated to be 5.9, 8.1 and 12.1Å, respectively.

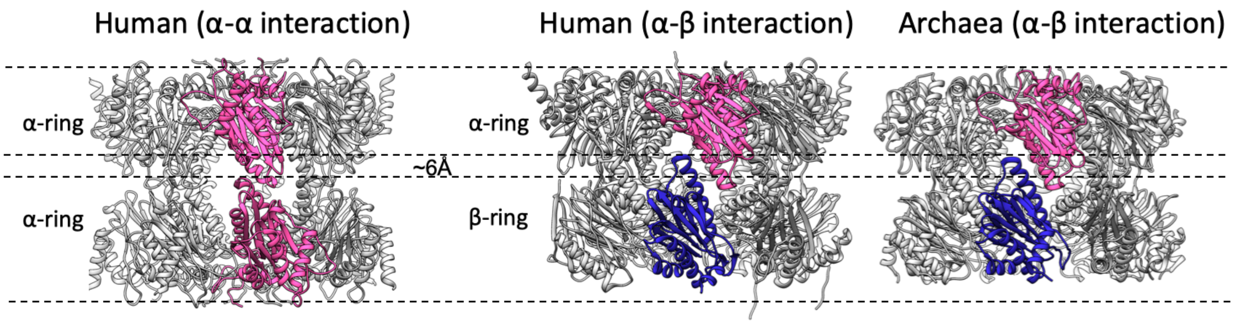

Supplementary Figure 2. Comparison of molecular interactions between the α-α ring of the symmetric-type α7 homo-tetradecamer and the α-β ring in the 20S core particle. The helix-helix interactions between α-β rings in the 20S core particles of human (PDB ID: 6RGQ, cryo-EM structure) and archaea (PDBID: 6UTF, cryo-EM structure) are deeper by approximately 6 Å than that of α-α ring in the symmetric-type α7 homo-tetradecamer. Pink: α subunit, Blue: β subunit.

Supplementary Table S1. Statistics for Cryo-EM imaging, data processing and refinement of model.

Data Collection			
Electron microscopy	JEM-2200FS		
Camera	DE20		
Voltage	200 kV		
Magnification	40,000		
Calculated pixel size	1.42 Å		
Exposure per frame (s)	0.2		
Dose per second (e-/Å2/s)	8		
Electron dose	40 electron/Å^2		
Number of frames	25		
Defocus range	1.0 - 3.0 μm		
**Image Processing**			
Frame alignment	MotionCor2		
CTF estimation software	CTFFIND 4.1.5		
Number of micrographs	100		
3D map reconstruction software	Relion 3.1		
Initial number of particles	107,079		
Type	Symmetric	Dislocated	Open
Particles contributing to final map	18,388	16,558	1,234
Applied symmetry	D7	C1	D1
Applied B-factor	-531 Å^2	-445 Å^2	-675 Å^2
Global resolution (FSC = 0.143)	5.9 Å	8.1 Å	12.1 Å
EMDB number	EMD-30990	EMD-30991	EMD-30992
**Model Building**			
Modeling software	*Coot, Phenix*		
Number of residues built	244		
R.m.s. deviation (bonds)	0.004		
R.m.s. deviation (angles)	0.9		
Ramachandran outliers	0.4 %		
Rotamer outliers	0 %		
Clashscore, all atoms	11.32		
PDB ID	7E55		

Supplementary Video S1. Structural fluctuation model of the α7 homo-tetradecamer in solution.
